# Retinal microvascular density analysis in patients with rheumatoid arthritis treated with hydroxychloroquine

**DOI:** 10.1007/s00417-022-05946-6

**Published:** 2022-12-29

**Authors:** Eliane Luisa Esser, Julian Alexander Zimmermann, Jens Julian Storp, Nicole Eter, Nataša Mihailovic

**Affiliations:** 1 Department of Ophthalmology, University of Muenster Medical Center, Muenster, Germany; 2 Medical Faculty OWL, Bielefeld University, Campus Klinikum Bielefeld, Bielefeld, Germany

**Keywords:** OCTA, HCQ retinopathy, Retinal thickness, Vessel density

## Abstract

**Purpose:**

Rheumatoid arthritis (RA) is the most common inflammatory joint disease, and hydroxychloroquine (HCQ) is an established treatment. The extent to which HCQ impacts ocular microvascular vessel density (VD) in patients with RA without evidence of HCQ retinopathy has not yet been conclusively clarified. The main aim of this study was to evaluate VD measured by optical coherence tomography angiography (OCTA) in patients with RA treated with HCQ.

**Methods:**

The VD data of the 3 × 3 mm OCT angiogram (RTVue XR Avanti, Optovue Inc., Fremont, California, USA) as well as the retinal thickness (RT) data of patients with RA (*n* = 30) and healthy controls (*n* = 30) were extracted and analyzed. The study group was further divided into patients undergoing HCQ treatment for > 5 years (high-risk-group) and < 5 years (low-risk group).

**Results:**

Patients with RA showed no evidence of VD reduction compared to the control group in all obtained regions (*p* > 0.05). Correlation analysis revealed no dependency between VD, RT, and HCQ therapy duration or cumulative HCQ dose (*p* > 0.05). High-risk patients showed a decreased VD in the superficial quadrant of the superficial capillary plexus compared to low-risk-patients (*p* = 0.022). Whole-en-face RT was reduced in the high-risk group compared to the control group (*p* = 0.019).

**Conclusion:**

Our study showed no evidence that HCQ diminishes VD in patients with RA without HCQ retinopathy measured by OCTA. However, RA patients with a long duration of therapy showed a significantly reduced RT. Our results suggest that quantitative VD analysis by OCTA may not be suitable for early detection of HCQ retinopathy and that the focus on detecting early HCQ retinopathy should be on intensive and sequential OCT diagnostics.

**Supplementary Information:**

The online version contains supplementary material available at 10.1007/s00417-022-05946-6.


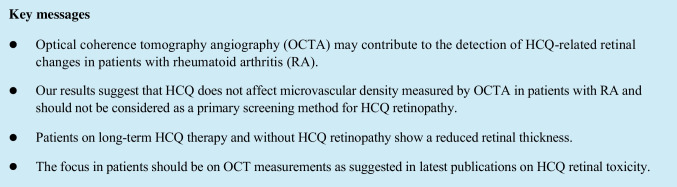


## Introduction

Rheumatoid arthritis (RA) is a chronic inflammatory autoimmune disease characterized by persistent synovitis, systemic inflammation, and autoantibodies production, such as rheumatoid factor and anticitrullinated peptides, and can affect a variety of tissues [1]. Although RA primarily affects the joints, it is known to frequently affect extra-articular structures including, but not limited to, the heart, vascular structures, and kidneys [1, 2]. RA is conventionally treated with a group of drugs known as disease-modifying anti-rheumatic drugs (DMARDs), which include glucocortidocids, methotrexate, leflunomide, sulfasalazine, and hydroxychloroquine (HCQ) [2]. HCQ is a well-known therapy able to prevent autophagosome–lysosome fusion, which is a critical step in the pathomechanism of rheumatic diseases [3]. This property allows considering HCQ as one of the major drugs used in autoimmune rheumatic conditions, especially in patients with systemic lupus erythematodes [4]. However, in patients with RA, antimalarials including HCQ are mainly reserved for patients with mild RA, as no new evidence regarding a good efficacy of hydroxychloroquine was found for RA in general and the historic studies had shown only weak clinical and no structural efficacy [5]. Nonetheless, there is still a large proportion of patients with RA being treated with HCQ. Although HCQ is generally well-tolerated, it might cause irreversible visual loss due to retinal toxicity. The risk of toxicity at recommended doses up to 5 years of treatment is < 1%, but it increases sharply after approximately 5 years of therapy and an overall prevalence of 7.5% was identified in patients taking HCQ for greater than 5 years, rising to almost 20% after 20 years of treatment [6, 7]. Early detection of HCQ retinopathy is crucial, since its damage to the retinal pigment epithelium (RPE) is irreversible and can progress even after cessation of the therapy [7]. The revised recommendations on screening for chloroquine and HCQ retinopathy of the American Academy of Ophthalmology from 2016 include automated visual fields plus spectral-domain optical coherence tomography (SD-OCT) as primary screening tests for detection of early HCQ retinopathy. Additional useful objective screening tests are multifocal electroretinogram (mfERG) providing objective corroboration for visual fields as well as fundus autofluorescence (FAF) showing topographical damage. [7]

In 2020, the Royal College of Ophthalmologists (RCOphth) also published revised recommendations on monitoring for chloroquine and HCQ stating that Humphrey visual field testing is no longer recommended as a first-line monitoring test, but that monitoring for HCQ retinopathy should involve SD-OCT imaging and widefield FAF imaging for all patients, if available [8]. These revised recommendations in recent years show that early detection of HCQ retinopathy remains challenging and new developments in multimodal imaging should be investigated for potential suitability as an early detection tool.

Optical coherence tomography angiography (OCTA) provides a three-dimensional visualization of choroidal and retinal microvascular layers as well as the possibility of quantification of the vessel density (VD) of macular capillary plexuses in a non-invasive way [9].

Different study groups demonstrated VD alterations in patients under HCQ treatment including patients with RA compared to healthy controls using OCTA [10–13]. These studies, however, show different, partly contradictory results with either reduced or increased VD and with the alterations occurring in different capillary plexus and regions. Also, they either included patients with other connective tissue diseases like systemic lupus erythematodes (SLE), did not analyze choriocapillary VD, or did not perform a correlation analysis between VD data and cumulative HCQ dose or duration of HCQ therapy [10–13].

Thus, the aim of the present OCTA study was to evaluate retinal and choriocapillary VD differences in patients with RA undergoing HCQ therapy in comparison to healthy controls and to correlate the data with the cumulative HCQ dose and duration of HCQ therapy.

## Methods

30 eyes of 30 patients with RA and 30 eyes of 30 gender and age matched healthy controls were consecutively included in this study. The healthy controls were recruited from subjects who visited our outpatient clinic for routine examinations and had no ophthalmological or systemic diseases. The study was approved by the Ethics Committee of the University of Muenster, North Rhine Westphalia, Germany. Before performing any examination, the study protocol was explained in detail and all participants signed an informed consent form. The study adhered to the tenets of the Declaration of Helsinki. Patients and controls with media opacities preventing high-quality imaging, vitreoretinal disease, previous retinal surgery, macular edema, glaucoma, or neurological disease were excluded from the study. All study participants underwent an ophthalmic examination including anterior segment examination, binocular fundus examination, and OCTA imaging. In the patient group, 10–2 visual field testing (standard automated perimetry), SD-OCT using the Spectralis OCT (Heidelberg Engineering, Germany), FAF, and multifocal electroretinogram were performed to rule out HCQ associated retinopathy. Only patients with no signs of HCQ toxicity were included in this study. Patients treated with HCQ for > 5 years were classified as the “high-risk group” (*n* = 21); patients with a HCQ treatment for < 5 years were classified as the “low-risk group” (*n* = 9). This classification was based on the revised recommendations on screening for HCQ therapy of the American Academy of Ophthalmology on screening for chloroquine and HCQ retinopathy which define a duration of use > 5 years as a factor of increasing risk of HCQ-retinopathy [7]. Other major risk factors like renal disease, tamoxifen use, excessive daily dose by weight, and retinal and macular diseases were defined as exclusion criteria. For the correlation analysis of the VD, RT, and the cumulative dose of HCQ, cumulative dose data were extracted from all patient records (*n* = 30).

### Optical coherence tomography angiography

OCTA imaging was performed using the RTVue XR Avanti system with AngioVue (Optovue Inc, Fremont, California, USA). Split-spectrum amplitude-decorrelation angiography (SSADA) was used to extract the OCTA information. OCTA imaging technology has been described in detail elsewhere [9]. The macula was imaged using a 3 × 3 mm scan. The automated segmentation was checked by an expert examiner before data analysis. All examinations were performed under the same conditions at the same location by an expert examiner. Images with poor signal strength (signal strength index (SSI) < 6) were not included in the study. In case of sufficient image quality of both eyes, the selection of the eye was randomised. Otherwise, the eye with the better SSI was selected. After imaging, the VD in SCP and DCP OCT angiogram, the RT, and the FAZ were analyzed using the integrated device software (AngioAnalytics, version 2017.1.0.151, Optovue Inc, Fremont, California, USA). To determine VD values of the choriocapillaris, OCTA images were exported. Using Adobe Photoshop CS6 (Adobe Systems, Inc., California), images were converted into grey scales. Each pixel was attributed to a value that represents the strength of the decorrelation signal. VD in the choriocapillaris was calculated as the mean decorrelation value of all pixels in the images (arbitrary unit, AU) [14].

### Statistics

IBM SPSS® Statistics 28 for Windows (IBM Corporation, Somers, NY, USA) was used for statistical analyses. The data was tested for normality distribution using the Shapiro–Wilk test, and all data did fit a normal distribution. The data are therefore presented as mean value ± standard deviation. The control and study group were compared using the two-sided independent sample *t*-test for normally distributed variables, and the degree of correlation between two variables was expressed as the Pearson’s correlation coefficient (*r*). For evaluating differences between the three subgroups (low-risk group, high-risk group, and control group), a closed testing procedure was used. Specifically, a one-way analysis of variance (ANOVA) was performed followed by a post hoc analysis via two-sided independent sample *t*-tests. Inferential statistics are intended to be exploratory (hypothesis-generating), not confirmatory, and are interpreted accordingly. The comparison-wise type-I error rate is controlled instead of the experiment-wise error rate. The chosen level of significance was *p* < 0.05.

## Results

There was no significant difference in age between RA patients and healthy controls (*p* = 0.203) and between patients of the high-risk group and the low-risk group (*p* = 0.192). The demographic characteristics of patients and healthy controls are shown in Table 1. For the comparisons of HCQ therapy duration and cumulative doses between the low-risk group and the high-risk group the values of Hedges *g* were 1.271 and 1.007, which puts our data into the moderate to large effect sizes. It is about the equivalent of ROC AUCs of 0.815 and 0.762 [15].

**Table 1:** Demographic data of study population. Statistically significant differences are marked in bold. Low-risk-group: rheumatoid arthritis (RA) patients treated with HCQ < 5 years. High-risk-group: RA patients treated with HCQ > 5 years

	Control group	Study group	*p*-value	Low-risk group	High-risk group	*p*-value
*n*	30	30	-	9	21	-
Gender f/m	27/3	27/3	-	8/1	19/2	-
Mean age (years) ± SD	53.4 ± 9.9	57.5 ± 14.2	0.203	51.7 ± 14.9	59.9 ± 12.9	0.192
Mean duration of HCQ therapy (months) ± SD	-	90.70 ± 58.72	-	38.44 ± 20.31	113.10 ± 55.49	** < 0.001**
Mean cumulative dose of (g) ± SD	-	760 ± 581	-	350 ± 242	937 ± 599	** < 0.001**

*SD* standard deviation, *HCQ* hydroxychloroquine, *g* gram, *f* female, *m* male

**Fig. 1:**
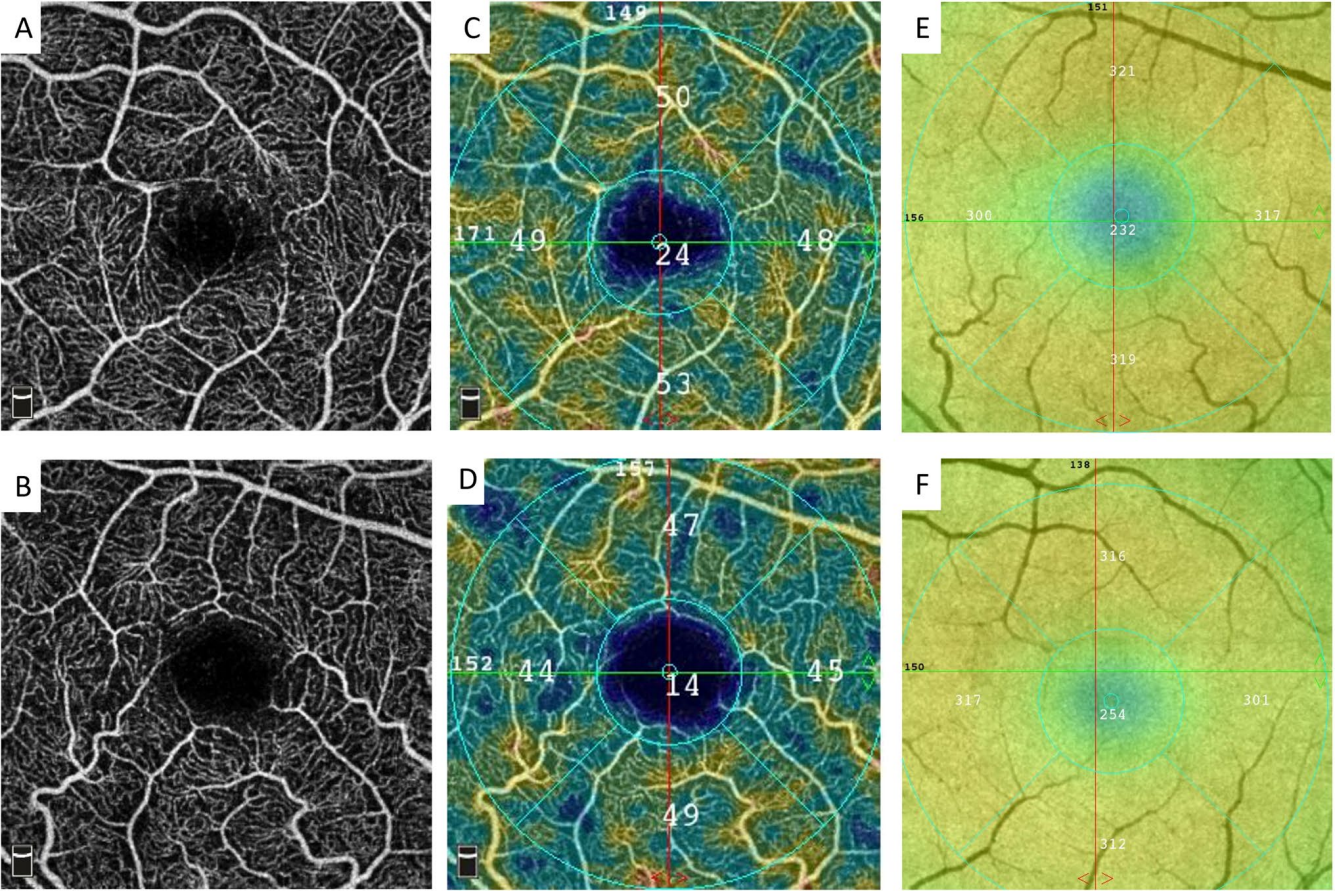
**A**–**F** Optical coherence tomography angiography (OCTA) and retinal thickness (RT) map. Exemplary 3 × 3 mm OCTA images of the macula (**A**, **B**: *whole-en-face* image of the superficial capillary plexus; **C**, **D** color-coded images indicating the analyzed regions) and RT map (**E**, **F**) of a patient with rheumatoid arthritis (top row) and an age-matched healthy control (bottom row)

VD analysis showed no statistically significant differences of the VD data between the study group and control group (*p* > 0.05) (Fig. 1; Table 2). Correlation analysis between the HCQ therapy duration, cumulative HCQ dose, and VD or retinal thickness data revealed no statistically significant dependency, either (*p* > 0.05) (Supplemental Table 1).

**Table 2:** Vessel density (VD, %) data of the rheumatoid arthritis (RA) group and control group obtained in the regions indicated, as well as the foveal avascular zone area (FAZ) and retinal thickness (RT) data. Data are presented as mean ± standard deviation. A *t*-test was used to compare both groups. Bold: statistically significant differences. SCP = superficial retinal capillary plexus; DCP = deep retinal capillary plexus; CC = choriocapillaris; SSI = signal strength index

	OCTA	RA group (*n* = 30)	Control group (*n* = 30)	*p*-value
SCP (VD)	Whole-en-face	45.5 ± 2.98	45.39 ± 2.66	0.887
	Fovea	19.61 ± 6.95	20.72 ± 5.61	0.498
	Parafovea	47.80 ± 3.99	47.94 ± 2.76	0.879
	Superior-hemi	47.21 ± 4.79	47.57 ± 3.02	0.725
	Inferior-hemi	48.37 ± 3.43	48.34 ± 2.67	0.964
	Temporal	46.71 ± 3.63	46.04 ± 3.59	0.476
	Superior	48.37 ± 4.74	48.98 ± 3.17	0.565
	Nasal	46.56 ± 4.83	47.17 ± 2.55	0.546
	Inferior	49.63 ± 3.94	49.65 ± 2.64	0.979
DCP (VD)	Whole en Face	50.25 ± 3.55	49.84 ± 3.89	0.676
	Fovea	35.47 ± 8.80	36.66 ± 7.00	0.562
	Parafovea	52.99 ± 3.31	51.23 ± 5.05	0.116
	Superior-hemi	53.10 ± 3.40	51.57 ± 3.79	0.103
	Inferior-hemi	52.67 ± 3.48	51.66 ± 3.81	0.292
	Temporal	53.62 ± 3.47	51.81 ± 3.72	0.057
	Superior	52.76 ± 3.70	51.29 ± 4.06	0.149
	Nasal	53.34 ± 3.84	51.87 ± 3.76	0.140
	Inferior	52.25 ± 3.52	51.39 ± 4.06	0.386
CC (VD)	Whole en face	114.29 ± 5.60	115.97 ± 4.91	0.221
FAZ (mm^2^)		0.24 ± 0.11	0.23 ± 0.10	0.924
RT (µm)	Whole-en-face	306.30 ± 13 .74	314.43 ± 11.63	**0.016**
	Fovea	263.73 ± 23.30	262.37 ± 17.91	0.800
	Parafovea	320.67 ± 12.95	321.20 ± 20.76	0.905
	Superior-hemi	321.07 ± 13.78	324.83 ± 12.35	0.269
	Inferior-hemi	320.47 ± 12.29	323.70 ± 12.11	0.309
	Temporal	312.27 ± 13.33	315.03 ± 11.49	0.393
	Superior	327.07 ± 13.94	327.47 ± 12.89	0.331
	Nasal	324.77 ± 14.38	325.03 ± 12.41	0.163
	Inferior	321.87 ± 11.79	325.03 ± 12.41	0.315
SSI		8.1 ± 0.9	8.0 ± 0.8	0.554

The *whole-en-face* (WEF) retinal thickness, however, was significantly reduced in the study group compared to healthy controls (*RT*_*WEF*_ study group: 306.30 µm ± 13.74 µm, *RT*_*WEF*_ control group: 314.43 µm ± 11.63 µm, *p* = 0.016; Table 2). An analysis of the three groups low-risk, high-risk, and control group showed that the WEF-RT was only significantly reduced in the high-risk-group (*RT*_*WEF*_ high-risk-group: 305.62 µm ± 14.19 µm, *RT*_*WEF*_ control group: 314.43 µm ± 11.63 µm, *p* = 0.019; *RT*_*WEF*_ low-risk-group: 307.89 ± 13.32 µm, *RT*_*WEF*_ control group: 314.43 µm ± 11.63 µm, *p* = 0.160).

A comparison of the VD data between the low-risk-group and the high-risk-group revealed a significantly reduced VD in the superior quadrant of the SCP in the high-risk-group (*VD*_*SCP sup*_ low-risk group: 50.55% ± 1.52%, *VD*_*SCP sup*_ high-risk-group: 47.44% ± 5.35%, *p* = 0.022) and a non-significant trend in the parafoveal area of the SCP (*VD*_*SCP parafovea*_ low risk group: 47.02% ± 4.36%, *VD*_*SCP parafovea*_ high-risk-group: 49.63% ± 2.16%, *p* = 0.1) (Table 3).

**Table 3:** Vessel density (VD, %) data of the patient subgroups obtained in the regions indicated as well as the foveal avascular zone area (FAZ) and retinal thickness (RT) data. Data are presented as mean ± standard deviation. A *t*-test was used to compare both groups. Bold: statistically significant differences. SCP = superficial retinal capillary plexus; DCP = deep retinal capillary plexus; CC = choriocapillaris; low-risk-group: rheumatoid arthritis (RA) patients treated with HCQ < 5 years; high-risk-group: RA patients treated with HCQ > 5 years

	OCTA	Low-risk group (*n* = 9)	High-risk-group (*n* = 21)	*p*-value
SCP (VD)	Whole-en-face	46.35 ± 2.44	45.13 ± 3.16	0.315
	Fovea	20.45 ± 7.70	19.25 ± 6.78	0.673
	Parafovea	49.63 ± 2.16	47.02 ± 4.36	0.100
	Superior-hemi	49.46 ± 2.16	46.24 ± 5.31	0.093
	Inferior-hemi	49.83 ± 2.37	47.75 ± 3.66	0.128
	Temporal	48.25 ± 2.38	46.06 ± 3.91	0.132
	Superior	50.55 ± 1.52	47.44 ± 5.35	**0.022**
	Nasal	48.41 ± 3.45	45.77 ± 48.87	0.174
	Inferior	51.38 ± 2.38	48.87 ± 4.28	0.112
DCP (VD)	Whole-en-face	50.72 ± 3.58	50.04 ± 3.60	0.639
	Fovea	36.19 ± 7.68	35.16 ± 9.41	0.774
	Parafovea	52.82 ± 3.46	53.06 ± 3.32	0.860
	Superior-hemi	53.22 ± 3.52	53.04 ± 3.44	0.904
	Inferior-hemi	52.40 ± 3.55	52.78 ± 3.53	0.789
	Temporal	53.17 ± 3.12	53.81 ± 3.67	0.652
	Superior	52.79 ± 3.90	52.74 ± 3.71	0.975
	Nasal	53.33 ± 3.40	53.35 ± 4.10	0.990
	Inferior	52.00 ± 3.85	52.36 ± 3.46	0.806
CC (VD)	Whole-en-face	113.84 ± 7.86	114.48 ± 4.54	0.779
FAZ (mm^2^)		0.24 ± 0.11	0.23 ± 0.12	0.862
RT (µm)	Whole-en-face	307.89 ± 13.32	305.62 ± 14.19	0.686
	Fovea	259.67 ± 20.81	265.48 ± 24.56	0.541
	Parafovea	320.11 ± 12.25	320.90 ± 13.53	0.881
	Superior-hemi	321.22 ± 12.68	321.00 ± 14.52	0.969
	Inferior-hemi	319.00 ± 11.81	321.10 ± 12.72	0.676
	Temporal	311.33 ± 13.76	312.67 ± 13.47	0.807
	Superior	324.00 ± 12.49	324.10 ± 14.82	0.987
	Nasal	324.22 ± 12.65	325.00 ± 15.35	0.895
	Inferior	320.33 ± 11.19	322.52 ± 12.24	0.649

## Discussion

In this quantitative OCTA study, our results suggest no VD differences between RA patients undergoing HCQ therapy and healthy controls. Particularly, we could not show a correlation between the VD and cumulative HCQ dose or HCQ therapy duration. Previous OCTA studies in the literature show different results and conclusions on the influence of HCQ on VD [11–13]. Hence, the impact of HCQ on VD as well as the relevance of OCTA diagnostics in these patients is not yet fully clarified. Details of the patient characteristics and the main results of the aforementioned studies are summarized in Table 4.

**Table 4:** Overview and characteristics of the studies which compared microvascular vessel density (VD) data in patients with rheumatoid arthritis (RA) and healthy controls (CTR) using optical coherence tomography angiography (OCTA)

Publication	Patients/controls (*n*)	Included diseases (*n*)	HR/LR (*n*)	Age (mean ± SD)	HCQ therapy duration (mean ± SD)	Cumulative HCQ dose in g (mean ± SD)	Analyzed parameters/OCTA device	Correlation analysis	HCQ retinopathy	Results
Goker et al. (2018)	20/20	SLE (unknown) RA (unknown)	HR only	HR: 55.58 ± 8.33 CTR: 54.61 ± 8.62	HR: 70.25 ± 10.17 (months)	Not specified	VD (SCP, DCP) FAZ RTVue XR Avanti**	No	No	- Reduced VD in HCQ group (region: SCP fovea, DCP fovea)
										- Enlarged FAZ area in HCQ group
Ozek et al. (2018)	40/20	RA (40)	HR (24) LR (16)	HR: 45.47 ± 12.4 LR: 38.38 ± 15.4 CTR: 42.06 ± 8.31	HR: 123.9 ± 21.5 LR: 22.81 ± 10.5 (months)	HR: 520.34 ± 112.71 LR: 330.12 ± 138.56	VD (SCP, DCP) FAZ RT RTVue XR Avanti**	No	No	- Reduced VD in HR vs. CTR (region: DCP temporal, DCP hemi-inferior)
										- Reduced RT in patients vs. CTR (region: SCP inferior, SCP temporal, DCP hemi-inferior)
Iacono et al. (2021)	16/16	RA (16)	n/a	RA: 61.2 ± 12 CTR: 59.2 ± 12	treatment naïve	Treatment naïve	VD (SCP, DCP) FAZ RTVue XR Avanti**	n/a	n/a	- Reduced VD in RA group vs. CTR (region: SCP global area, SCP inner ring, SCP superior)
Sargues et al. (2022)	51/25	SLE (21) RA (17) SS (3) CTD (10)	All: HR (22) LR (29) *RA: HR (2) LR (15)*	HR: 53.37 ± 12.57 LR: 53.51 ± 10.25 CTR: 55.05 ± 13.60	HR: 12.00 ± 4.32 LR: 2.57 ± 1.58 (years)	Not specified	VD (SCP, MCP, DCP, TCP, CC) FAZ DRI OCT Triton Plus***	No	No	- Increased VD in HR vs. CTR (region: SCP nasal, parafoveal, temporal; MCP superior, nasal, parafoveal, total; TCP inferior, total)
										- Reduced CMT in HR vs. CTR
*Esser et al. (2022)	30/30	RA (30)	HR (21) LR (9)	HR: 59.9 ± 12.9 LR: 51.7 ± 14.9 CTR: 53.4 ± 9.9	HR: 113.10 ± 55.49 LR: 38.44 ± 20.31 (months)	HR: 937 ± 599 LR: 350 ± 242	VD (SCP, DCP, and CC) FAZ RT *RTVue XR Avanti* **	Yes	No	- No reduced VD in RA patients vs. CTR
										- No correlation between VD and HCQ duration/cumulative dose
										- reduced VD in HR vs. LR (region: SCP superior)
										- Reduced WEF RT in HR vs. CTR

*HCQ* hydroxychloroquine; *SLE* systemic lupus erythematosus; *SS* Sjögren’s syndrome; *CTD* connective tissue disease; *CC* choriocapillaris; *SCP* superficial capillary plexus; *DCP* deep capillary plexus; *MCP* middle capillary plexus; *TCP* total capillary plexus; *FAZ* foveal avascular zone; *WEF* whole-en-face; *RT* retinal thickness; *CMT* central macular thickness; *n* number of included subjects; *LR* low-risk group, RA patients treated with HCQ < 5 years; *HR* high-risk group, RA patients treated with HCQ > 5 years

^*^Present study; **Optovue Inc, Fremont, CA; ***Topcon, Tokyo, Japan

Our results are in line with those of Remoli Sargues et al., who demonstrated even an increase of VD in patients with various autoimmune diseases who underwent HCQ treatment and therefore suggest that HCQ retinal toxicity is not vascular mediated [13]. However, they only included 17 patients with RA and only 2 of them belonged to the high-risk group. In contrast to that, Ozek et al. included 40 patients with RA and showed a reduced VD in the deep temporal and deep hemi-inferior vascular plexus in the high-risk group, suggesting a relevance of OCTA measurements to the early findings of HCQ toxicity [11].

In our analysis, there was no difference between the VD data of the control group and high-risk-group neither in the superficial nor in the deep vascular plexus. However, VD values in the deep retinal layer show a weaker repeatability compared to the VD values of the superficial retinal layer [16] and OCTA imaging in the deep vascular layer is more challenging and affected by artefacts [17, 18].

Other studies showing a reduction of the VD using OCTA in patients under HCQ treatment included patients with various diseases and did not differentiate between patients with RA and other autoimmune diseases, such as SLE which is known to be associated with immune complex-mediated microangiopathy and might cause retinopathy itself [13]. We believe it is very important to create homogenous study populations in these kind of quantitative OCTA studies, since it cannot be concluded from these findings whether the observed alterations were genuinely caused by the underlying disease or if these changes could be influenced or biased by the HCQ therapy. Moreover, as suggested in other OCTA studies on patients with SLE, HCQ might have a protective role on preserving microvasculature in autoimmune diseases while reducing the disease activity [13, 19].

To the best of our knowledge, none of the mentioned studies analyzing solely RA patients performed a correlation analysis between VD data and cumulative HCQ dose or HCQ therapy duration (Table 4). Although one might assume that a high cumulative dose or long therapy duration has an impact on VD, we did not find a tendency in our correlation analysis. Since none of our patients showed signs of HCQ retinopathy, we postulate that neither the cumulative HCQ dose nor the HCQ therapy duration do have an impact on VD in these patients and might not be suitable for determination of early retinopathy in these patients.

When comparing VD data of low-risk and high-risk patients, VD was significantly reduced in the superior quadrant of the high-risk group. We believe this result should be taken with caution and not equated with very early alterations/thinning of retinal structures by OCT analysis which suggest the inferior quadrant to be affected earlier than the superior quadrant [20, 21].

In patients with long HCQ therapy duration, we demonstrated a reduced whole-en-face RT compared to the control group. Latest publications of Kim et al. and Marmor et al. emphasize the sensitivity of RT analysis in detecting HCQ retinopathy [22–24]. Marmor et al. even suggest that sequential RT analysis might be the earliest diagnostic sign of HCQ retinopathy [24]. In our study, population without clinical evidence for HCQ retinopathy, only the whole-en-face RT showed a significant difference between the high-risk-group and the healthy control group [23]. Due to the recent findings of the publications mentioned above, this could lead to the conclusion that this might be a very early sign for clinically not yet detected retinopathies [22–24].

Our study has some limitations worth noting: First, it is a cross-sectional study. Therefore, we cannot comment on the value of VD measurements for evaluation of disease progression. Further studies on OCTA imaging in RA patients undergoing HCQ treatment with a longitudinal design should be performed in future. A second limitation is the small sample size of the low-risk group. It might be that some of the negative findings (i.e. lack of significant differences) were due to the limited sample size. However, correcting for small cohort size with Hedges *g* still revealed moderate to large effect sizes. Moreover, compared to other OCTA studies evaluating VD in RA patients, our study has one of the largest numbers of RA patients with a long duration of therapy and high cumulative doses. In addition, the patients in the current study accounted a rather homogeneous age- and gender-matched study population.

In conclusion, the results of our study show no sign of reduction of microvascular density in patients with RA regardless of the duration of HCQ therapy. Moreover, VD data did not correlate with the duration of therapy or cumulative dose. Since the existing literature on VD alterations in patients undergoing HCQ treatment show very controversial results, we suggest that VD analysis using OCTA seems not to be a suitable additional diagnostic mean in detecting early signs of HCQ retinopathy in patients with RA. Considering the latest study results on RT diminution in patients with HCQ therapy in the literature and our presented results, the main focus on detecting early HCQ retinopathy should remain on intensive and sequential OCT diagnostics.

## Supplementary Information

Below is the link to the electronic supplementary material.

Supplementary file1 (DOCX 20 KB)

## Data Availability

All data of this study are available from the corresponding author upon reasonable request.
