# Supplementary material for: Retinal microvascular density analysis in patients with rheumatoid arthritis treated with hydroxychloroquine
Source: Graefes Arch Clin Exp Ophthalmol. 2022 Dec 29;261(5):1433–42. doi: 10.1007/s00417-022-05946-6 (PMC10148761; doi:10.1007/s00417-022-05946-6)
Supplement: Supplementary file 1 — Supplementary file1 (DOCX 20 KB) [file 417_2022_5946_MOESM1_ESM.docx]

**Supplementary Table 1.** Correlation analysis between optical coherence tomography angiography (OCTA) parameters (vessel density, *%*) and clinical parameters (cumulative dose, *g*; duration of hydroxychloroquine therapy, *months*) of the study group (n = 30).

*r = Pearson correlation coefficient; SCP = superficial capillary plexus; DCP = deep capillary plexus;*

*CC = choriocapillaris*

| **OCTA parameter** | **clinical parameter** | **r** | **p-value** |
| --- | --- | --- | --- |
|  |  |  |  |
| SCP whole en face | HCQ therapy duration | 0.138 | 0.468 |
| SCP fovea | HCQ therapy duration | 0.171 | 0.367 |
| SCP parafovea | HCQ therapy duration | -0.054 | 0.777 |
| SCP superior hemi | HCQ therapy duration | -0.088 | 0.644 |
| SCP inferior hemi | HCQ therapy duration | -0.075 | 0.693 |
| SCP temporal | HCQ therapy duration | -0.126 | 0.507 |
| SCP superior | HCQ therapy duration | -0.042 | 0.827 |
| SCP nasal | HCQ therapy duration | 0.001 | 0.996 |
| SCP inferior | HCQ therapy duration | -0.038 | 0.842 |
| DCP whole en face | HCQ therapy duration | 0.126 | 0.508 |
| DCP fovea | HCQ therapy duration | 0.211 | 0.264 |
| DCP parafovea | HCQ therapy duration | 0.167 | 0.377 |
| DCP superior hemi | HCQ therapy duration | 0.010 | 0.957 |
| DCP inferior hemi | HCQ therapy duration | -0.030 | 0.877 |
| DCP temporal | HCQ therapy duration | 0.079 | 0.677 |
| DCP superior | HCQ therapy duration | 0.254 | 0.175 |
| DCP nasal | HCQ therapy duration | 0.156 | 0.412 |
| DCP inferior | HCQ therapy duration | 0.104 | 0.586 |
| CC | HCQ therapy duration | 0.012 | 0.950 |
| RT whole en face | HCQ therapy duration | 0.003 | 0.988 |
| RT fovea | HCQ therapy duration | 0.182 | 0.337 |
| RT parafovea | HCQ therapy duration | 0.270 | 0.149 |
| RT superior hemi | HCQ therapy duration | 0.275 | 0.141 |
| RT inferior hemi | HCQ therapy duration | 0.287 | 0.125 |
| RT temporal | HCQ therapy duration | 0.253 | 0.177 |
| RT superior | HCQ therapy duration | 0.271 | 0.147 |
| RT nasal | HCQ therapy duration | 0.311 | 0.095 |
| RT inferior | HCQ therapy duration | 0.261 | 0.164 |
| FAZ | HCQ therapy duration | -0.186 | 0.325 |
|  |  |  |  |
| SCP whole en face | cumulative dose | 0.225 | 0.232 |
| SCP fovea | cumulative dose | 0.084 | 0.660 |
| SCP parafovea | cumulative dose | 0.122 | 0.519 |
| SCP superior hemi | cumulative dose | 0.070 | 0.713 |
| SCP inferior hemi | cumulative dose | 0.140 | 0.460 |
| SCP temporal | cumulative dose | 0.032 | 0.867 |
| SCP superior | cumulative dose | 0.117 | 0.538 |
| SCP nasal | cumulative dose | 0.169 | 0.372 |
| SCP inferior | cumulative dose | 0.142 | 0.453 |
| DCP whole en face | cumulative dose | 0.86 | 0.650 |
| DCP fovea | cumulative dose | 0.099 | 0.601 |
| DCP parafovea | cumulative dose | 0.073 | 0.701 |
| DCP superior hemi | cumulative dose | -0.079 | 0.679 |
| DCP inferior hemi | cumulative dose | -0.111 | 0.559 |
| DCP temporal | cumulative dose | -0.017 | 0.928 |
| DCP superior | cumulative dose | 0.156 | 0.410 |
| DCP nasal | cumulative dose | 0.060 | 0.754 |
| DCP inferior | cumulative dose | 0.051 | 0.788 |
| CC | cumulative dose | 0.127 | 0.503 |
| RT whole en face | cumulative dose | 0.020 | 0.918 |
| RT fovea | cumulative dose | 0.070 | 0.713 |
| RT parafovea | cumulative dose | 0.157 | 0.406 |
| RT superior hemi | cumulative dose | 0.167 | 0.378 |
| RT inferior hemi | cumulative dose | 0.179 | 0.345 |
| RT temporal | cumulative dose | 0.145 | 0.445 |
| RT superior | cumulative dose | 0.150 | 0.429 |
| RT nasal | cumulative dose | 0.221 | 0.241 |
| RT inferior | cumulative dose | 0.138 | 0.466 |
| FAZ | cumulative dose | -0.093 | 0.626 |

**Title: Retinal microvascular density analysis in patients with rheumatoid arthritis treated with hydroxychloroquine**

Eliane Esser, Julian Zimmermann, Jens-Julian Storp, Nicole Eter, Nataša Mihailovic*

Department of Ophthalmology, University of Muenster Medical Center, Muenster, Germany

Department of Ophthalmology, Klinikum Fulda, University of Marburg, Campus Fulda, Fulda, Germany

*Email: [nat.mihailovic@gmail.com](mailto:nat.mihailovic@gmail.com)
